# Dance’s Veterinary Tablet

**Published:** 1891-04

**Authors:** 


### REVIEWS.

DANCE'S VETERINARY TABLET. Wm. R. Jenkins, New York, 1891.

The tablet of A. A. Dance, copyright 1890, is a synopsis of the diseases of horses, cattle and dogs with the causes, symptoms and cures. It contains a tabulated sheet giving the part affected, diseases, causes, symptoms in horses, symptoms in cattle, symptoms in dogs, cures in horses, cures in cattle, and cures in dogs. Divided into external or local diseases of the skin, of the body generally, head, withers, udder, legs, hock, cannon, fetlock, and feet; internal diseases, system generally, organs of respiration, stomach, bowels and liver, kidneys and bladder, brain, eyes, throat and mouth. To these is added a series of some thirty prescriptions to govern these diseases.

This chart furnishes a general outline of the diseases of animals which will allow the owner, who has studied it, to select a specialist of practitioners who should be called in to aid him with the knowledge which he does not possess himself.

